# Mobilising the alumni of a Master of Public Health degree to build research and development capacity in low- and middle-income settings: The Peoples-uni

**DOI:** 10.1186/s12961-015-0064-1

**Published:** 2015-12-01

**Authors:** Richard F Heller, Pasipanodya I Machingura, Baba M Musa, Paramita Sengupta, Puja Myles

**Affiliations:** People’s Open Access Education Initiative (Peoples-uni), Eccles, Manchester, United Kingdom; College of Health Sciences, University of Zimbabwe, Harare, Zimbabwe; Department of Medicine, Bayero University, Aminu Kano Teaching Hospital, Kano, Nigeria; Christian Medical College and Hospital, Ludhiana, India; Faculty of Medicine & Health Sciences, University of Nottingham, Nottingham, United Kingdom

**Keywords:** Alumni, Low- and middle-income countries, Public health, Research capacity

## Abstract

**Background:**

Peoples-uni (People’s Open Access Education Initiative) was established to help build Public Health capacity in low- and middle-income countries (LMICs) through postgraduate level online courses. Graduates are invited to join a virtual alumni group. We report the results of efforts to meet the need for health research capacity building by exploring how the course alumni could be mobilised to perform collaborative research into the health problems of their populations.

**Methods:**

Two online surveys of Peoples-uni graduates were conducted with graduates from the first two and first four cohorts in 2013 and 2014, respectively, to explore the formation of an alumni group that would collaborate to further the research and development agenda in LMICs. This was followed by feedback on research-related activity and outcomes via the online alumni and tutors’ forum to estimate early indicators of alumni success in relation to capacity building in both the conduct and utilisation of research.

**Results:**

Responses were received from 26 (87% response rate) graduates of the first survey and 42 (60% response rate) of the second survey. Overall, 92% of the respondents to the first survey supported the creation of an alumni group, especially if it helped to develop their own research skills and improve the health of their populations. Findings from the second survey showed that study with Peoples-uni was felt to have had a major or potential impact on the careers of the respondents, with 19% of graduates having progressed to a PhD programme to further their research skills, and a further 48% being in the process of applying or intending to apply for doctoral studies. Further feedback shows that at least one collaborative study has been completed and published by alumni members with other collaborative studies planned. Ongoing support has been provided to graduates to help them publish their work and apply for individual or collaborative research grants.

**Conclusions:**

Harnessing the alumni of a Masters level course to perform collaborative research has considerable potential to build research capacity in LMICs.

## Background

Most of the world’s health research is performed in high-income countries with a focus on health concerns that affect such populations [1]. Moreover, interventions proven to work in the developed world may not be easily transferable to developing world contexts [2] and even research based in low- and middle-income countries (LMICs), but without local stakeholder involvement, may not reflect the country’s health priorities [3]. There is a growing expressed need to undertake research to address the major health inequities that remain between rich and poor countries [4] and, ideally, this research needs to be based in LMICs and carried out in collaboration with researchers based in these settings. Locally-led research in LMICs and its publication may be more likely to effect change in clinical practice than research performed in other settings [5]. However, LMICs generally have poorly developed research infrastructures [6] and often lack the data and the capacity to use them [7]. It is thus critical to build researcher capacity and infrastructure in low-income settings. While educational programmes targeting individuals within these settings will help to build such capacity [8], there is little information on the role that the graduates of such programmes play in meeting the research needs of their populations.

The People’s Open Access Education Initiative, Peoples-uni, is an open access education initiative (http://peoples-uni.org) established to help build public health capacity in LMICs [9]. The initial activity has been to develop and offer postgraduate level courses available either as stand-alone courses for continuing professional development or leading to a Master of Public Health (MPH) award. Two groups of modules are available – Foundation Sciences for Public Health (including skills in research methodology) and Public Health problems challenging LMIC populations. The MPH dissertation requires a literature review and the development of a research project protocol. The courses are all taught online by an international group of volunteer tutors and this, as well as the use of open educational resources, allows the courses to be offered at very low cost. The online, flexible, and part-time nature of the programme, spread over at least 3 years, allows students to remain in their usual post and workplace in their country. The majority of students and graduates are currently working as health professionals in LMICs; hence, personpower is not depleted during study, and graduates can apply their new skills for the benefit of their populations immediately. Students are encouraged to use real-world problems encountered by them in their work setting as the subject of both the coursework (for example, developing an evidence-based intervention or evaluating an intervention) and the dissertation. Courses run to a semester timetable, and the use of online discussion forums in each module, as well as in the Students Corner (to which all students are enrolled), encourages students to network with each other, providing a familiar platform for students to continue to communicate after graduation.

The mission statement of Peoples-uni is “*To contribute to improvements in the health of populations in low- to middle-income countries by building Public Health capacity via e-learning at very low cost*” [10]. Among the six main objectives is: “*Work with the graduates of the educational programme, and other relevant partner organisations, in teaching, research, implementation of evidence based health policy and advocacy to improve the health of their populations*” [10]. In order to meet this goal, an Alumni group was established. Restricted to those with an MPH (to ensure that members have a high degree of knowledge and skills), graduates are automatically enrolled in the Alumni group and given access to a dedicated space on the password-protected courses web site (http://courses.peoples-uni.org). The site includes a list of the objectives of the Alumni group, links to relevant resources and organisations, and a series of discussion forums covering the major objectives of the group (collaborative research and education) as well as a general discussion forum. It is overseen and discussions are facilitated by one of the Peoples-uni leadership group members, with other tutors and external experts invited to join for particular projects or mentoring of individuals. The organisation Statistics without Borders (http://community.amstat.org/statisticswithoutborders/home) has also agreed to provide statistical advice for projects.

The first group of students graduated with an MPH in 2012, and they and each subsequent graduating cohort were enrolled in the Alumni group.

The purpose of this paper is to report the results of efforts to develop health research capacity and enhance collaboration in research via the alumni network of an online open access education initiative.

## Methods

Two surveys were conducted with members of the Alumni group. The first was to determine their research capacity building needs and how to best fulfil these via the group’s activities. The second was to assess the career impact of their studies to that date. Questions were developed in discussion with a small group of graduates and tutors. Both used anonymised online survey forms, with one general reminder to the group. Survey 1 was administered to the first two graduating cohorts in August 2013, 2 and 8 months after becoming an alumni, and Survey 2 was administered to the graduates of the first four graduating cohorts, in December 2014, between 6 months and 2 years after becoming an alumni. Both surveys used open source survey tools, with links sent to each of the Alumni group members who were registered at that time. Descriptive statistics were used and results tabulated as numbers and percentages.

In addition, alumni and tutors were asked, through discussion forums both in the Alumni group site and on the Tutors Corner discussion form, to provide feedback on research-related activities and outcomes to provide any early indicators of alumni success in relation to capacity building in both the conduct and utilisation of research.

## Results

The first graduates of the course graduated in 2012 with an MPH granted by our partner, Manchester Metropolitan University, and, to date, there are 88 Alumni group members (Table 1 represents the regions from which the alumni come).

**Table 1:** **Region from which alumni come – to May 2015 (n = 88)**

**Country**	**Number (%)**
Southern Africa	13 (15%)
Eastern Africa	22 (25%)
West Africa	29 (33%)
Indian subcontinent	13 (15%)
Elsewhere	11 (12%)

The characteristics of the first 117 students to enrol in the MPH programme (entry is restricted to those who pass two Peoples-uni modules initially at the Masters level) are shown in Table 2. Around 40% have a medical degree and/or work in public health and/or have a previous Diploma, Masters or PhD award. Stated motivation on application was mainly the desire for personal career development or to help improve the health of their populations.

**Table 2:** **Characteristics of first 117 Masters of Public Health (MPH) enrolments**

**Characteristic**		**Number**
Date of birth	Before 1970	26
	1970–1979	53
	1980 or later	36
Qualification	Non-health degree	21
	Non-medical health degree	35
	Medical degree	49
	Other	11
Higher degree	Certificate	13
	Diploma	27
	Masters	23
	PhD	3
Occupation	Academic	7
	Clinical (non-public health)	28
	Other health	19
	Public health	50
	Other	11

### Surveys of alumni

#### Survey 1 findings (first two graduating cohorts)

Responses were received from 26 of the 30 (87%) graduates; 24 of the 26 (92%) respondents supported the creation of an Alumni group, especially if it helped to develop their own research skills and improve the health of their populations (Table 3).

**Table 3:** **Answers to question: “Do you think it is a good idea to have an Alumni group?” (n = 26 respondents)**

**Five possible options offered**	**Number (%)**
No	0
Yes, if it helps me to continue to develop my personal knowledge and skills	24 (92%)
Yes, if it helps me perform research which will help improve the health of my population	24 (92%)
Yes, if it helps develop opportunities and skills for teaching	22 (85%)
Yes, if it helps me get onto a PhD programme	19 (73%)

Respondents were asked to rank the potential activities of the group in order of preference, from 11 possible options. The top ranked request was for resources for further study followed by the development of collaborative research opportunities (Table 4). Support for admission to a PhD programme was ranked fourth, and 25 of the 26 (96%) respondents answered ‘Yes’ to the question: “If we set up a pre-PhD programme which identified skills needed to prepare and apply for a PhD programme, would you be interested to join?” (Peoples-uni does not have the resources to offer a PhD itself). Further, 22 of the 26 (85%) respondents answered ‘Yes’ to the question: “Would you be happy to act as a Peoples-uni tutor (or continue in this role if you have already started)”.

**Table 4:** **Answers to the question: “Which of these activities do you think would be good to include in the Alumni group. Please rank in order of importance with 1 being the most important.” (n = 26 respondents)**

**Option (in order of the question)**	**Overall rank achieved by each option**
Adding resources for further study	1
Starting new courses for further study	5
Developing collaborative research for the group as a whole	2
Developing collaborative research between just a few members	6
Developing collaborative research with Peoples-uni tutors	3
Discussions on teaching methods	7
Development of collaborative teaching programmes	8
Identifying individual mentors for each of the alumni	9
Identifying mentors for groups of alumni	10
Helping to get onto a PhD programme	4
A ‘Helpdesk’ by Peoples-uni tutors or others for advice	11

In free text responses, four of the alumni stated that they were already teaching research methods or performing their own research.

#### Survey 2 findings (first four graduating cohorts)

Responses were received from 42 of 70 (60%) graduates. In response to the question: “How much impact has your study with Peoples-uni had on your career?”, 6 (14%) respondents answered that “It was the cause of my getting a new job” and a further 6 (14%) answered “It was the cause of my promotion” (multiple answers were allowed). An additional 21 (50%) answered “Nothing yet, but I expect it will”. Additionally, 8 (19%) graduates said they had already joined a PhD programme in answer to the question: “Have you enrolled in a PhD or other higher degree programme after Peoples-uni?”, and a further 20 (48%) had applied or planned to apply.

Among the 20 free-text comments to the question “Please give any examples of a new job or promotion resulting from your Peoples-uni experience”, early outcomes related to capacity building in both the conduct and utilisation of research emerged.

“*I am glad to say that I am already studying for my PhD in the UK. It’s not easy to gain admission into PhD program in the world leading universities but I was admitted into a number of them. Thanks to Peoples-uni.*”

“*Through Peoples-uni I have been able to perform my new job well. I currently coordinate Operational Research activities with a lot of confidence and competency as an alumni of Peoples-uni.*”

“*The program – mainly the resources – helped me to gain knowledge and develop skills in maternal and child health. As a result I was able to obtain a new position with an NGO.*”

“*I am currently using the knowledge and experience of tutoring with the local University and currently am supervising six master students.*”

Below are three examples of the impact of Peoples-uni on each of three alumni (co-authors of this paper).

PIM: Coordinated a multicentre collaborative study leading to peer-reviewed publication. Improved supervision of student research projects. Invitation to a United Kingdom University for research supervision training.

BMM: Obtained place in a United States University research grant writing programme. Two peer-reviewed publications (systematic reviews). Runs weekly epidemiology education sessions for interns to help build research capacity in Nigeria.

PS: Selected to be a Principal Investigator for three Indian Council of Medical Research funded projects, one national task force, and Co-investigator in a WHO study. Reviewer for international and national journals. Collaboration on international research grant applications and projects with other Peoples-uni tutors.

### Use of the site by alumni

A number of discussions have taken place on the site, and these have all been related to the goals of the Alumni group rather than social networking. The site facilitator has posted information about news and further educational opportunities. A journal club to discuss papers of potential interest was not joined by the majority and was discontinued. As ideas for collaborative research projects are identified, a forum is established for each one and interested participants enrolled. More than one third of the alumni (37%) have actually posted at least once to one or more of the discussion forums.

### Collaborative research among alumni

To date, the group has designed and completed one research project which has been published in a peer-reviewed journal [11]. The idea for the study came from a discussion between one of us (RFH) and a scientist external to the group. The study itself was designed, analysed and written for publication by three of the alumni together with the two originators, and involved a survey of the value of the use of information technology in the implementation of guidelines to improve clinical or public health practice. None of the participants had previously performed research together, and 44 of the possible 48 members of the Alumni group at the time participated in the data collection. Other collaborative studies are planned, amongst the alumni and between alumni and tutors, including developing protocols on non-communicable disease, HIV/AIDS and national health insurance. One of the alumni has taken responsibility as research coordinator for the group and alumni are leading each of the discussions about new projects. The group has recently been given access to an online data collection tool [12], which will stimulate further collaborative research project developments.

### Indirect effects

A number of tutors have given individual advice to graduates to help them publish their work and apply for individual or collaborative research grants. The role of a tutor for Peoples-uni is to be the facilitator of an online discussion forum on one of the five topics in a module, and some tutors also mark assignments and act as individual project supervisors during the Dissertation. To date, 14 of the alumni have been accepted as tutors (the requirement is possession of an MPH and demonstration of competence as a co-tutor). The majority of tutors are from high-income countries, so the addition of those from LMICs countries adds a valuable dimension to the programme. A further four alumni are acting as Student Support Officers – whose role is to guide students through the complexities of the course requirements and practice. A number of the alumni are cascading their knowledge to others through their own local teaching, and are applying for research grants themselves and with colleagues. Much of this is work in progress although research publications by alumni are beginning to emerge [13-15].

## Discussion

Peoples-uni has, from the start, had a very clear remit to see beyond the award of the academic degree to improve the health of LMIC populations via increased health research capacity. The ongoing facilitation of graduates to tackle these health problems using an evidence-based approach has a number of advantages. The graduates are proven to have postgraduate level research and critical appraisal skills, they are used to working collaboratively via online discussions where they are asked to respond to each other’s postings as part of the educational process, and they have established links with many of the tutors who have specialised skills and an international perspective. Most alumni are already working as health professionals, and therefore have a practical understanding of local health problems, which, combined with research skills, provides an excellent platform to perform healthcare-related research and development projects of importance to their populations. Many are working in health departments and thus will have the opportunity to apply research findings to health policy. The support and collaboration from other alumni and Peoples-uni tutors offer continuing opportunities for collaboration and skills development.

There was a high demand for further study to the PhD level, and for a PhD preparation programme. While Peoples-uni does not have the scope to offer a PhD programme, a number of the alumni have already found one. Further exploration of how to prepare graduates for further study and help with identifying PhD opportunities, particularly those allowing study at a distance, should be a priority in order to build further research capacity and build on the results of a Masters level programme.

As time goes on, we hope to be able to assess outcomes of research projects conducted, translation of research findings into policy to deal with important health problems, papers published, and teaching programmes developed. Some of this work will, itself, require infrastructure funding, but some is already underway, with some early successes within the existing resources of the Peoples-uni initiative and its alumni group. It will be important for the alumni to access, or develop, local supportive environments for their research. Our online mentorship programme should be seen as an enabling process and not one that can replace the benefits of local support. To help facilitate this, we ask each student to identify a local adviser during the Dissertation phase, both to ensure that the project planned there is locally relevant, but also to promote local linkages.

The alumni have been enthusiastic about continuing their involvement with Peoples-uni after graduation – there has been good interest and involvement in the Alumni site. It has not been as easy to encourage leadership to emerge from the group, although this is lately beginning to happen. The impact of the Peoples-uni project as a whole will depend on the alumni performing research education and advocacy to impact on health policy, and we hope that the creation and facilitation of the Alumni group will assist in achieving this goal. While we hope this model will deliver on increased research capacity in LMIC settings, we currently do not have sufficient data to evidence this; nevertheless, early indicators of success include the willingness of the graduates to engage with the Alumni group and to participate in collaborative research projects. The willingness of many of the tutors to continue to collaborate, mentor and advise, and of the alumni to join the tutor group themselves, also suggests that mutual support will continue and develop into a collaborative and continuing force for research in LMICs. The methods of facilitating and supporting this development continue to be explored and developed. In addition to the educational programme gaining from the alumni through new tutors and Student Support Officers, individual tutors have also gained benefit from this collaboration – as an example, one tutor involved three of the alumni as tutors in an online course on global health that she ran for United Kingdom medical students.

This paper has a number of limitations, including the relatively early stage at which we report the findings, the few concrete results of collaborative research, and the short time over which the alumni group has been in existence. Attrition over time, failure to identify resources for continuing research, and lack of the development of leadership from among the alumni will be threats to ultimate success.

Future evaluations of the Alumni group network will include longer-term survey-based follow-up of alumni (metrics such as enrolment for further study, involvement in research projects, research grant applications, publication in peer-reviewed journals, academic career pathways etc.) as well as in-depth qualitative research that could provide insight into the pathways between the course itself, the alumni network and capacity building.

## Conclusions

Harnessing the alumni of a Masters level course to perform collaborative research has considerable potential to build research capacity in LMICs. Peoples-uni provides a web-based platform and training for virtual collaboration among alumni and tutors that crosses geographical barriers. The opportunities created by this network of diverse alumni to carry out multi-country studies and interventions has already demonstrated preliminary feasibility, and provides a platform for future collaboration and the generation of new knowledge for health research and development.
